# Post cardiac arrest care and follow-up in Sweden – a national web-survey

**DOI:** 10.1186/s12912-016-0123-0

**Published:** 2016-01-09

**Authors:** Johan Israelsson, Gisela Lilja, Anders Bremer, Jean Stevenson-Ågren, Kristofer Årestedt

**Affiliations:** Department of Internal Medicine, Division of Cardiology, Kalmar County Hospital, SE-39185 Kalmar, Sweden; Department of Medical and Health Sciences, Division of Nursing Science, Linköping University, SE-58185 Linköping, Sweden; Kalmar Maritime Academy, Linnaeus University, SE-39182 Kalmar, Sweden; Department of Clinical Science, Division of Neurology, Lund University, Lund, Sweden; Department of Neurology and Rehabilitation Medicine, Skane University Hospital, SE-22185 Lund, Sweden; Faculty of Caring Science, Work Life and Social Welfare and the Centre for Prehospital Research, University of Borås, SE-50190 Borås, Sweden; Division of Emergency Medical Services, Kalmar County Hospital, SE-39185 Kalmar, Sweden; Information School, University of Sheffield, Regent Court, 211 Portobello Street, Sheffield, S1 4DP England; eHealth Institute, Linnaeus University, SE-39182 Kalmar, Sweden; Center for Collaborative Palliative Care, Linnaeus University, SE-39182 Kalmar, Sweden

**Keywords:** Heart arrest, Survivors, Family members, Follow-up, Quality of life, Guidelines

## Abstract

**Background:**

Recent decades have shown major improvements in survival rates after cardiac arrest. However, few interventions have been tested in order to improve the care for survivors and their family members. In many countries, including Sweden, national guidelines for post cardiac arrest care and follow-up programs are not available and current practice has not previously been investigated. The aim of this survey was therefore to describe current post cardiac arrest care and follow-up in Sweden.

**Methods:**

An internet based questionnaire was sent to the resuscitation coordinators at all Swedish emergency hospitals (*n* = 74) and 59 answers were received. Quantitative data were analysed with descriptive statistics and free text responses were analysed using manifest content analysis.

**Results:**

Almost half of the hospitals in Sweden (*n* = 27, 46 %) have local guidelines for post cardiac arrest care and follow-up. However, 39 % of them reported that these guidelines were not always applied. The most common routine is a follow-up visit at a cardiac reception unit. If the need for neurological or psychological support are discovered the routines are not explicit. In addition, family members are not always included in the follow-up.

**Conclusions:**

Although efforts are already made to improve post cardiac arrest care and follow-up, many hospitals need to focus more on this part of cardiac arrest treatment. In addition, evidence-based national guidelines will have to be developed and implemented in order to achieve a more uniform care and follow-up for survivors and their family members. This national survey highlights this need, and might be helpful in the implementation of such guidelines.

**Electronic supplementary material:**

The online version of this article (doi:10.1186/s12912-016-0123-0) contains supplementary material, which is available to authorized users.

## Background

Every year, approximately 275 000 persons in Europe suffer from an out-of-hospital cardiac arrest (OHCA) [1] whereas the number of in-hospital cardiac arrest (IHCA) is not known. Recent decades have shown major improvements in survival rates and in Sweden more than 1 000 persons survive cardiac arrest (CA) annually [2]. Most CAs are caused by a cardiovascular disease [2] and survivors are at risk of suffering cardiac complications [3]. Survival may also be associated with neurological impairments due to the lack of oxygen to the brain at the time of the arrest. Severe brain injuries in survivors are uncommon but mild to moderate cognitive impairments, e.g., memory problems have been reported in as many as 30–50 % of the survivors [4, 5]. Additionally, psychological impairments may be present [6]. Surviving a life-threatening event such as CA will affect the lives of both survivors and their family members [7, 8]. Following a near death experience, survivors may become more aware of their vulnerability. Family members can be forced to confront feelings of unreality, uncertainty, hopelessness and, in addition, they can experience feelings of inadequacy and an overwhelming responsibility in the situation [7]. Moreover, patients and family members are at risk of psychological stress due to the critical illness *per se*. It is well-known that patients recovering from critical illness and intensive care are at risk for psychological problems such as anxiety, depression and post-traumatic stress disorders [9], which may affect the patient’s ability to perform activities in everyday life and participate in society.

Psychological problems, cognitive dysfunction and difficulties in performing activities of daily life have been associated with decreased health among CA survivors [10]. A review article concludes that health and quality of life (QoL) among survivors appears to be acceptable or good, but also reports major variations between different studies and within study populations [11]. Some studies report that suffering a CA has negative effects on QoL, and that survivors have poorer QoL compared to a normal population [10, 12, 13]. Other studies have not been able to show any differences [14–16].

In order to address problems caused by CA and to support health among survivors and their family members, structured post CA care is needed. Today, national guidelines for post CA care and follow-up programs are not available in Sweden. The Swedish Resuscitation Council (SRC) has recommended an information package for survivors and their family members since 2011 [17]. This material contains information about CA in general and stories of experiencing CA, told by survivors and their family members, in particular. However, the success of the implementation is unknown. Patients suffering CA are often admitted to intensive care units (ICU) [2]. In Sweden, patients with critical illness in general participate in follow-ups performed by intensive care nurses post ICU discharge. However, these follow-ups have been described as varying extensively in design and not being available for all [18]. The goal of the ICU follow-up is to promote the patients’ recovery by focusing on three domains: the past, the present and the future. The past aims to support patients’ understanding, the present includes actual physical, cognitive and psychological status, and the future includes rehabilitation or other interventions to promote health. The last step has been the weakest point so far in Scandinavia [18], where other countries promote more structured guidelines for rehabilitation, as in the UK with the National Institute for Health and Clinical Excellence (NICE) guidelines [19]. Whether these ICU follow-ups include the majority of CA survivors is unknown.

Since cardiac etiology is common [2], CA survivors are likely to receive cardiovascular follow-up, primarily focused on physiological secondary prevention [20]. However, because they are at risk of also suffering neurological and emotional complications [5, 6], which might affect their QoL [10], specific care and follow-up is necessary [6, 21–23]. Previous research describing specific post CA care and follow-up is sparse [24–27]. In many countries, including Sweden, national guidelines for post CA care and follow-up programs are not available, and current practice has, to our knowledge, not previously been investigated. The aim of this survey was therefore to describe current post CA care and follow-up in Sweden.

## Methods

### Design

This national survey had a descriptive cross-sectional design. The overall theoretical rationale for this study was based on a perspective of health as a multidimensional concept. Health can be enhanced over time, through a process supported by health care, especially regarding the development of coping strategies and learning within the family. Nursing is viewed as a science of health-promoting interactions: to actively promote patient and family strengths, to help them cope with a life-changing event, and to achieve life goals [28]. The study was designed and conducted in accordance with the World Medical Association Declarations of Helsinki [29] and Swedish Ethics Legislation concerning informed consent and confidentiality [30]. Formal ethical approval was not required, according to ethics legislation in Sweden (SFS 2003:460), since no sensitive personal information was collected and the participants answered in their role as health care professionals. Participation was voluntary and confidential, and participants were assured that hospitals and individuals would be impossible to trace in the published material. Informed consent was presumed if the participant chose to complete the questionnaire.

### Data collection

A study specific questionnaire was developed for this survey (Additional file1, English translation). The development was guided by a conceptual framework of health care quality, a comprehensive literature review, and the authors’ own experience. The framework of health care quality, described by Donabedian [31], comprises three components; structure, process and outcome. These three components cover: 1) contextual factors in which care is provided such as physical equipment, facilities, environments, human resources and organizational characteristics, 2) health care actions taken by professionals, patients and family members, and 3) effects of health care on patients, family members or populations. In developing the tool, all three components were included.

The initial version of the questionnaire included 11 closed-ended questions covering six topics: local guidelines (3 questions), routines for follow-up visits (3 questions), content of the follow-up visits (1 question), family involvement (1 question), patient reported outcome measures (PROMs) and information material (2 questions), and quality registry (1 question). To ensure content validity [32], an expert group including researchers, members of the SRC, health care professionals and a psychometrician evaluated the questionnaire. The expert group critically reviewed the questionnaire, which was then revised, guided by their comments. In addition to minor refinements, an open-ended question was added.

Thus, the final questionnaire had a total of 12 questions. Six questions were constructed as statements, e.g., “At my hospital we have explicit guidelines for post CA care”, answered by a Likert type scale with four response options: “Agree”, “Partly agree”, Disagree” or “Don’t know”. Four questions included possible content of post CA care and follow-up, and were constructed to be answered with “Yes”, “No” or “Don’t know”. One multiple choice question was posed to elucidate the timing of follow-up visits.

The final question had an open-ended format where respondents were given the opportunity to write their own comments, thoughts and/or proposals in relation to the previous questions and answers. This question aimed to provide supplementary information for a better understanding of the quantitative findings.

With assistance from the SRC, a web-based version of the questionnaire was sent out to the resuscitation coordinators at all Swedish emergency hospitals (*n* = 74) in January 2013. After 2 reminders, 59 answers (80 %) were received.

### Data analysis

Quantitative data were analysed with descriptive statistics, using STATA 13.1 for Mac (StataCorp LP, College Station, TX, USA). The qualitative free text responses (open-ended question) were independently analysed (by AB and JI) using manifest content analysis [33], aiming to describe and compare the respondents’ answers to the six topics in the questionnaire, to identify responses with similar content. The data were then read several times to become familiar with the content. The qualitative data were deductively grouped according to the topics, followed by inductive categorization and abstraction of the data. Finally, the findings were discussed between all researchers until agreement was reached.

## Results

Quantitative and qualitative results are reported together in connection with each topic. The categories and sub-categories, identified by manifest content analysis, are presented in Table 1.

**Table 1:** Categories and sub-categories based on manifest content analysis of answers to the open-ended question; “Do you have any other considerations or suggestions related to post cardiac arrest care and follow-up?”

Sub-category	Category
No follow up structure	Lack of guidelines
Instructions are missing	
Planning for care programs	
Varying time intervals	Varying routines
Different professionals involved	
Follow up based on needs	
Routines are missing	
Diagnosis guide follow up	Inexplicable differences
Cause of the CA guide follow up	
Type of hospital ward guide follow up	
Insufficient family follow up	Invited or forgotten
Lack of time	

### Local guidelines for post CA care – lack of guidelines

Almost half of the hospitals in Sweden (*n* = 27, 46 %) reported having local guidelines for post CA care. However, 39 % of these hospitals reported that guidelines were not always applied. More than half of the hospitals did not have local guidelines.

Open-ended responses revealed that a few participants (*n* = 2) were aware of this deficiency:

*“Unfortunately we do not have any guidelines.”*

One participant seemed to have become aware of the problem while answering the survey:

*“Here it seems like we need help to improve post cardiac arrest care. This survey made this very clear.”*

### Routines for follow-up visits – varying routines

**Fig. 1:**
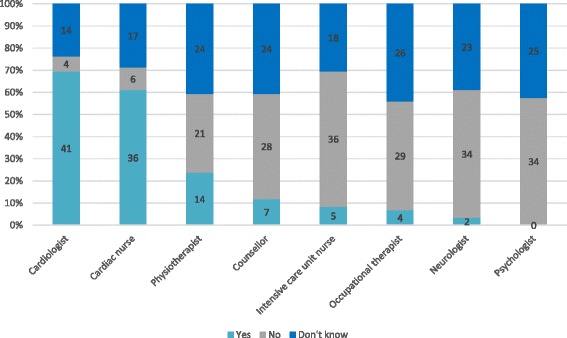
Health care professionals present at follow-up visits [*n* = 59]

**Fig. 2:**
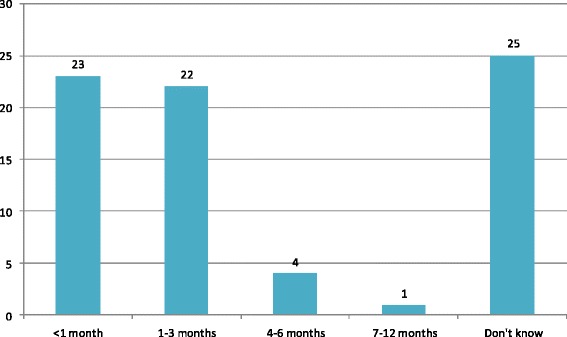
Time for follow-up visits [some hospitals offer more than one visit]

The most common routine was a follow-up visit at a cardiac reception unit to meet with a cardiologist (*n* = 42, 70 %) and/or a cardiac nurse (*n* = 36, 61 %) (Fig. 1). In general, the follow-up visits took place within one month (*n* = 23, 39 %) and/or within 3 months (*n* = 22, 37 %). However, 42 % (*n* = 25) did not know the time for the follow-up visits (Fig. 2). A minority of the hospitals reported to have routines for follow-up visits to other occupational categories; intensive care nurse (*n* = 5, 9 %), neurologist (*n* = 2, 3 %), counselor (*n* = 7, 12 %), occupational therapist (*n* = 4, 7 %) and physiotherapist (*n* = 14, 24 %) (Fig. 1).

Respondents’ comments in the open-ended question showed how the follow-up was organized regarding professionals involved (*n* = 4), and time intervals (*n* = 3):

*“At the cardiology section follow-up for cardiac arrest patients is always performed by a cardiologist eight weeks after discharge.”*

Sometimes respondents (*n* = 5) indicated that there was a need for follow-up involving other professionals with various expertise:

*“It would be good to have routine follow-up visits to e.g.: Neurologist, Occupational therapist, Counselor, Psychologist.”*

If the need for neurological or psychological support is discovered the routines are not explicit. One respondent indicated that this situation is not optimal:

*“There is a lot we could do for the survivors and their family members. For example, a follow-up visit to me. Unfortunately, there is not enough time. All the time is needed to make things work at the hospital with all the education.”*

### Content of the follow-up visits – inexplicable differences

**Fig. 3:**
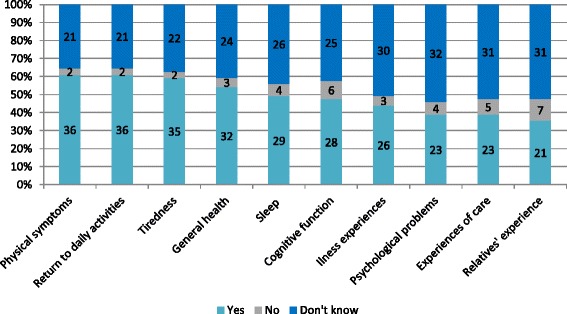
Content of the follow-up visits [*n* = 59]

The most common standardized content at the follow-up visits were; tiredness and fitness (*n* = 35, 59 %), physical symptoms (*n* = 36, 61 %), general health (*n* = 32, 54 %) and return to daily activities (*n* = 36, 61 %). In 48 % (*n* = 28) cognitive function and in 39 % (*n* = 23) psychological problems were followed up. The questions about the content of post CA care were frequently answered with “don’t know” (36–54 %) (Fig. 3).

In the open-ended question, respondents (*n* = 3) commented that the content of post CA care was dependent on care settings:

*“The post cardiac arrest care looks different depending on what ward the patients are admitted to.”*

According to a few respondents (*n* = 2), the content could also be dependent on aetiology:

*“The follow-up is dependent on the cause of cardiac arrest and may therefore vary quite a lot.”*

### Family involvement in post CA care – invited or forgotten

In total, 44 % (*n* = 26) of the hospitals had as a routine to involve family members by inviting them to participate in post cardiac arrest care and follow-up. Further, 17 % (*n* = 10) of the hospitals invited family members occasionally, but not as a routine. The rest of the hospitals had no such routine or did not know, 3 % (*n* = 2) and 36 % (*n* = 21) respectively.

In the open-ended question, one respondent emphasised the importance of routines for involving family members in post CA care and follow-up:

*“The patients and their family members meet our cardiac rehabilitation nurse two weeks after discharge. Also a follow-up visit to a cardiologist one month after discharge.”*

### Patient reported outcome measures and information material

A minority (*n* = 12, 20 %) of the hospitals used PROMs to detect problems experienced by the patients themselves, e.g., EuroQol-5 Dimensions, Short Form-36 and Visual Analogue Scale for well-being. The majority of the hospitals (*n* = 44, 75 %) in some way used the national information material from the SRC as a routine to support survivors and their family members.

### Quality registry in post CA care

In order to follow and evaluate CA care and follow-up, a majority of Swedish hospitals (*n* = 51, 86 %) report data to the Swedish registry for cardiopulmonary resuscitation. The registry is internet based and data are collected on three occasions: CA event, hospital discharge and 30 days post arrest [2].

## Discussion

Despite the need for structured post CA care for survivors and family members, few studies have described these aspects [24–27]. Overall, this survey showed that guidelines are not available at many hospitals in Sweden, and consist mainly of traditional cardiac rehabilitation with follow-up visits at cardiac reception units. Resuscitation coordinators in general lack knowledge about how post CA care is organized. Answers to the open-ended question confirm these findings. This raises the question of whether the hospitals meet post CA care needs, among survivors and family members, in order for health to be restored and improved over time.

Since cardiac follow-up does not include all CA patients and ICU follow-ups seem to be uncommon, there is no clear pathway for CA survivors and their family members. According to answers from the open-ended question, differences could depend on diagnosis, cause of CA and type of hospital ward. In addition, our results imply great variability in care between hospitals. In contrast to national intentions, striving for equal care [34], our findings showed that quality of post CA care and follow-up seems to depend on where the patient lives. This is not unique to Sweden and corresponds to the results of a Canadian study by Keenan, et al. [35]. In their study, regional differences in ICU care after CA were also described. However, the results entail the importance of local, national and international guidelines. Encouragingly, written information material with the aim to support patients’ and family members’ recovery seemed to be implemented as an element of post CA care at the majority of the Swedish hospitals, and therefore in some ways can help to create uniformity. Further, processes that promote families’ ability to cope with the life-threatening event might be strengthened by learning from the experiences of others [28].

As in previous investigations of post ICU care and follow-up [18], the content of the visits in our survey mainly included the present status of the patients (e.g. assessing current physical function, daily activities and health). The lack of routines on how to handle problems identified, shows low focus on rehabilitation in order to support and promote health and recovery over time. A randomized controlled follow-up intervention especially designed for CA survivors has been tested in the Netherlands [36]. This [37] is one of few health-promoting interventions intended for CA survivors and their caregivers, which have been described in detail. This individualized, semi-structured psychosocial intervention, ‘*Stand still…, and move on’*, is designed for early detection of emotional and cognitive problems, and for providing information and support. It also aims to promote self-management as well as an early referral to specialized care if needed. The intervention consists of one to six consultations conducted by specially trained nurses [36]. The recently published results showed that the intervention improved QoL and decreased anxiety among CA survivors at one-year post CA. However, it did not improve outcome for caregivers. These results are very likely to contribute to improvements in post CA care and follow-up [25].

Less than half of the hospitals reported that they had as a routine to invite family members to participate in post CA care. However, it remains unclear how and if concerns among family members are detected. Maybe they are invited to participate in follow-up, or maybe they are forgotten. Since mild cognitive dysfunction appears to be common among OHCA survivors [4, 5], and cognitive dysfunction among survivors has been shown to be associated with strain among family members [38], it is important for family members to be included in post CA care. In addition, stress, anxiety and decreased QoL among relatives have been reported [23]. As previous dyad studies show that patients and spouses affect each other’s health [39, 40], survivors and their family members will likely affect each other in the same way. There is also reason to assume that the function of the family is affected by, as well as affects, health and QoL among both patients and family members [41]. Family members might play an important part in the post CA care. Therefore, nursing should actively promote strengths and health among both survivors and their family members [28].

A minority of the hospitals used PROMs to detect problems among survivors. After this study was conducted, the Swedish registry for cardiopulmonary resuscitation began including PROMs in the follow-up, for example, health-related quality of life among survivors using questionnaires and telephone interviews. PROM data will contribute to better knowledge of the life situation among survivors, since the number of patients available for research will increase. This knowledge could constitute a starting point for the testing of screening methods and health promoting interventions as well as the creating of national guidelines. In addition, PROMs play a key role for person centred care, by influencing the care based on patient specific information [42]. In a recently published editorial, Smith and Bernard [16] highlight the need for more research to determine what outcome measures accurately describe obstacles important to patient- and family health after a CA event. They argue that good measurements, with the ability to capture predictors for poor health, could aim to target and evaluate interventions. Consensus has not been reached concerning what assessments to use to evaluate outcome after a CA. However, one of the most descriptive guidelines can be found in the recommendations of the American Heart Association from 2011 [43]. In a recently published study of 249 OHCA survivors [38] it was concluded that questionnaires and telephone interviews to assess cognitive function and QoL can be recommended for CA research.

In the present study, most of the follow-up visits took place within the first three months after the CA. However, a few open-ended responses indicated that follow up visits were sometimes based on patients’ needs. This might indicate a growing awareness of health as multidimensional [28], making the patient perspective essential. Further, there is a lack of knowledge and guidance about optimal timing and intervals for evaluating the patient’s QoL after a CA. The UK NICE guidelines for follow-up after a general critical illness suggest a structured pathway for assessments: at ICU stay (during the stay and before discharge), at ward-based care (during and before discharge), and at a follow-up visit 2–3 months after discharge [19]. A structured pathway for rehabilitation of present findings has further been suggested by Jones [9]. However, these guidelines are not designed especially for a CA group and most of the interventions still lack sufficient evidence. In addition, many survivors, especially those suffering IHCA, may not be admitted to an ICU at all.

Nurses, in particular those working at cardiac- and intensive care reception units, should be aware that CA patients and relatives could be at risk of not receiving optimal post CA care. Therefore, they should pay special attention to individual needs and possible health-problems. In order to improve post CA care and follow-up, future research should focus on the needs of CA survivors and their family members and on the testing of health promoting interventions. Such knowledge will be helpful for improving hospital care and developing guidelines.

### Limitations

Cross-sectional surveys with descriptive designs are beneficial approaches for health care researchers, particularly in new area of inquiry. However, they have some drawbacks that need to be considered [44]. The questionnaire was developed specifically for this study and had not undergone any extensive validation. However, the tool development was guided by a well-used conceptual framework about health care quality [31, 45]. Although the questions cover all three components, i.e. structure, process and outcome, not all aspects of these were included. This choice was made to make the questionnaire short and easy to complete. The open-ended question allowed respondents to provide supplementary answers to the closed-ended questions, as well as to express other aspects of post CA care. In addition, content validity was determined by a researcher with extensive experience in instrument development and psychometrics. The response rate was high, which indicated that the questions were easy to understand and complete. This also indicated an interest for the study. In addition to a high response rate, the respondents were well spread geographically and there were different types of cities, and small and large hospitals. Despite the high response rate, the findings should be interpreted and generalized with some caution.

Another limitation was sparse qualitative data from the open-ended question, reported by 15 of the respondents. For this reason, qualitative data were deductively grouped according to the six established topics, followed by inductive categorization and abstraction of the data. With more extensive material, it would have been preferable to start the analysis by coding the data. Despite this limitation, the qualitative data contributed to a better understanding of the quantitative findings.

In this study, we sent the questionnaire to resuscitation coordinators, since they were most likely to know the routines at the hospitals. However, in order to get more comprehensive results, answers from other groups, e.g., cardiac rehabilitation nurses or nurses responsible for post ICU care and follow-up, might also have been of interest. Another weakness is that we cannot say anything about which hospitals completed the questionnaire and which did not, since the answers were given anonymously. Still our results imply the need for improvements in post CA care, e.g., by finding structured pathways for referral and including other specialities, in order to increase the chances of promoting all aspects of health and QoL among survivors and their families, especially emotional and cognitive aspects.

## Conclusions

Although efforts have already been made to improve post CA care and follow-up, many hospitals need to focus more on this part of CA treatment. In addition, evidence-based national guidelines will have to be developed and implemented in order to achieve more uniform care and follow-up for survivors and their family members. This national survey highlights this need and might be helpful in the implementation of such guidelines.

## Additional file

Additional file 1: 
**English translation of the study specific questionnaire.** (DOC 42 kb)

## Abbreviations

CA: Cardiac arrest
IHCA: In-hospital cardiac arrest
ICU: Intensive care unit
NICE: National Institute for Health and Clinical Excellence
OHCA: Out-of-hospital cardiac arrest
PROMs: Patient reported outcome measures
SRC: Swedish Resuscitation Council
QoL: Quality of life
