# Supplementary material for: Post cardiac arrest care and follow-up in Sweden – a national web-survey
Source: BMC Nurs. 2016 Jan 9;15:1. doi: 10.1186/s12912-016-0123-0 (PMC4706707; doi:10.1186/s12912-016-0123-0)
Supplement: Additional file 1: — English translation of the study specific questionnaire. (DOC 42 kb) [file 12912_2016_123_MOESM1_ESM.doc]

**
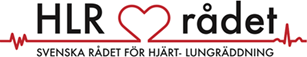
**

The Swedish Resuscitation Council

*Post cardiac arrest care and follow-up in Sweden*

*– a national web-survey*

1. **Does your hospital register data in the Swedish registry for cardiopulmonary resuscitation?**
   - Yes
   - No
   - Don´t know
2. **We have explicit guidelines for post cardiac arrest care and follow-up at my hospital**
   - Agree
   - Partly agree
   - Disagree
   - Don´t know
3. **In my opinion, these guidelines are used in clinical practice**
   - Agree
   - Partly agree
   - Disagree
   - Don´t know
   - We don´t have any explicit guidelines
4. **Which healthcare professionals will the cardiac survivor meet (as part of routine follow-up) BEFORE discharge from your hospital?**

Cardiac nurse

- - Yes
  - No
  - Don´t know

Intensive care unit nurse

- - Yes
  - No
  - Don´t know

Cardiologist

- - Yes
  - No
  - Don´t know

Neurologist

- - Yes
  - No
  - Don´t know

Counsellor

- - Yes
  - No
  - Don´t know

Psychologist

- - Yes
  - No
  - Don´t know

Occupational therapist

- - Yes
  - No
  - Don´t know

Physiotherapist

- - Yes
  - No
  - Don´t know

1. **Which healthcare professionals will the cardiac survivor meet (as part of routine follow-up) AFTER discharge from your hospital?**

Cardiac nurse

- - Yes
  - No
  - Don´t know

Intensive care unit nurse

- - Yes
  - No
  - Don´t know

Cardiologist

- - Yes
  - No
  - Don´t know

Neurologist

- - Yes
  - No
  - Don´t know

Counsellor

- - Yes
  - No
  - Don´t know

Psychologist

- - Yes
  - No
  - Don´t know

Occupational therapist

- - Yes
  - No
  - Don´t know

Physiotherapist

- - Yes
  - No
  - Don´t know

1. **When is the time for follow-up visits?** (choose all applicable alternatives)
   - Within 1 month
   - Within 3 months
   - Within 6 months
   - Within 12 months
   - Don´t know
2. **What is the content of the follow-up visits?** (choose all applicable alternatives)
   - Physical symptoms
   - Return to daily activities
   - Tiredness
   - General health
   - Sleep
   - Cognitive function
   - Illness experiences
   - Psychological problems
   - Relatives’ experiences
   - Experiences of care
3. **Relatives are routinely invited to follow-up visits at my hospital**
   - Agree
   - Partly agree
   - Disagree
   - Don´t know
4. **At my hospital, we routinely use the information material from the Swedish Resuscitation Council to support survivors and their relatives**
   - Agree
   - Partly agree
   - Disagree
   - Don´t know
5. **Does your hospital routinely follow-up patients elsewhere than in the Swedish registry for cardiopulmonary resuscitation?**
   - Yes
   - No
   - Don´t know
6. **If my hospital discovers problems or distress among survivors (or relatives), we have**

**routines for referral (i.e. Neurologist, Psychologist)**

- - Agree
  - Partly agree
  - Disagree
  - Don´t know

1. **Do you have any other considerations or suggestions related to post cardiac arrest care and follow-up?**

|  |
| --- |
|  |
|  |
|  |
|  |
|  |
|  |
|  |
|  |
|  |
|  |
|  |
|  |
|  |
|  |
|  |

Please check that you have answered all questions.

Thank you for participating!

If you have any questions please contact:

Johan Israelsson, RN, PhD-student, Resuscitation coordinator

County Council of Kalmar, Swedish Resuscitation Council

[johani@ltkalmar.se](mailto:johani@ltkalmar.se), +46480448646
